# Relationship between molecular properties and degradation mechanisms of organic solar cells based on bis-adducts of phenyl-C_61_ butyric acid methyl ester†

**DOI:** 10.1039/d1tc05768e

**Published:** 2022-04-25

**Authors:** Xueyan Hou, Andrew J. Clarke, Mohammed Azzouzi, Jun Yan, Flurin Eisner, Xingyuan Shi, Mark F. Wyatt, T. John S. Dennis, Zhe Li, Jenny Nelson

**Affiliations:** International Collaborative Laboratory of 2D Materials for Optoelectronics Science and Technology of Ministry of Education, Institute of Microscale Optoelectronics, Shenzhen University Shenzhen 518060 China; Department of Physics and Centre for Plastic Electronics, Imperial College London London SW7 2AZ UK jenny.nelson@imperial.ac.uk; SPECIFIC, Swansea University Bay Campus Swansea Wales SA1 8EN UK; National Mass Spectrometry Facility, Swansea University Medical School Singleton Park Swansea SA2 8PP UK; State Key Laboratory of Motor Vehicle Biofuel Technology, International Research Center for X Polymers, Department of Polymer Science and Engineering, Zhejiang University Hangzhou 310027 China; Haina-Carbon Nanostructure Research Center, Yangtze Delta Region Institute of Tsinghua University Jiaxing 314006 China; School of Engineering and Materials Sciences, Queen Mary University of London London E1 4NS UK zhe.li@qmul.ac.uk

## Abstract

Environmental stability remains a major challenge for the commercialisation of organic solar cells and degradation pathways remain poorly understood. Designing materials for improved device stability requires an understanding of the relationship between the properties of the donor or acceptor molecule and different degradation mechanisms. Here we study the correlations between various molecular parameters of the fullerene derivative bis-PCBM and the degradation rate of polymer:bis-PCBM organic solar cells, based on the same carbazole-*alt*-benzothiadiazole polymer, in aerobic and anaerobic conditions. We compare eight high purity bis-PCBM isomers with different electronic, chemical and packing properties along with PCBM and the mixture of bis isomers. In the case of aerobic photodegradation, we find that device degradation rate is positively correlated to the LUMO energy of the bis-PCBM isomer and to the degree of crystallinity of the isomer, while the correlation of degradation with driving force for epoxide formation is unclear. These results support the idea that in these samples, aerobic photodegradation proceeds *via* superoxide formation by the photogenerated polaron on the fullerene, followed by further chemical reaction. In the absence of air, photodegradation rate is correlated with molecular structure, supporting the mechanism of microstructural degradation *via* fullerene dimerization. The approach and findings presented here show how control of specific molecular parameters through chemical design can serve as a strategy to enhance stability of organic solar cells.

## Abstract 2

Change of the side chain position of bis-PCBM can modulate the molecular parameters and further serve as a strategy to control the stability of organic solar cells.

## Graphical abstract

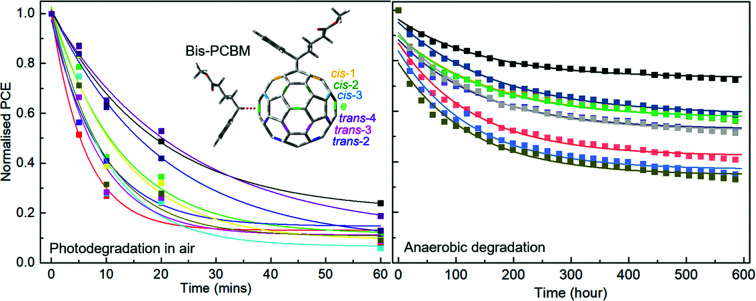

## Introduction

Environmental stability of solution-processed organic photovoltaic (OPV) devices remains the major challenge for their widespread application. Whilst remarkable progress has been made in improving both the efficiency and functional understanding of OPV devices, mainly due to advances in design of so-called non-fullerene acceptors (NFAs) of highly adaptable molecular structure, control of device stability remains a challenge. Overcoming the device stability barrier requires methods to identify the causes of degradation and to relate changes in device performance to specific degradation mechanisms and molecular design parameters.^1–3^

Prior studies of OPV device stability, many of them addressing material systems based on fullerene acceptors such as phenyl-C_61_-butyric methyl acid ester (PCBM), have revealed several different degradation mechanisms. These include chemical degradation of the organic semiconductor by photo-oxidation,^4,5^ chemical degradation of the electrodes^6–9^ and structural disintegration of the donor: acceptor blend by thermally induced phase separation^10–12^ or, in the case of polymer:fullerene blends, *via* the photo-induced oligomerisation of fullerenes under operation in light and inert atmosphere.^13–16^ Despite persistent research interest in the mechanisms that control OPV efficiency, design rules to guide the enhancement of OPV material and device stability are still lacking, mainly due to the poor fundamental understanding of their degradation mechanisms. Some recent studies have attempted to extract general trends from available data. For example, some evidence suggests a relationship between the acceptor's electron affinity and the device photostability in air.^17^ Other studies suggest a relationship between degree of crystallinity in the acceptor domain and photo-oxidation rate.^18,19^ Whilst these observations suggest that it may be possible to relate the mechanisms of degradation to molecular parameters, such as chemical structure, thermodynamic stability, electronic energy levels, strain, or crystallinity, the correlation between such molecular parameters and device degradation behaviour has not yet been well studied.^14^ It is difficult to carry out systematic molecular property – device stability investigations using NFAs since the chemical structures are diverse and there is, as yet, limited knowledge of their properties within blend films. Fullerene derivatives, in contrast, are ideal as a model molecular system, since they offer a high degree of chemical and morphological similarity whilst allowing systematic variations of their electrochemical properties and chemical structure. Fullerenes remain of interest for the understanding of both organic solar cells, where they serve as an electron acceptor, and perovskite solar cells where they serve as electron transport layer material.^17,20,21^

Higher adduct fullerenes, where multiple similar side chains are added to the same fullerene cage, have received limited attention for application in OPV, partly because the presence of multiple isomers and the resulting energetic and structural disorder introduced by multiple side-chain positions on the fullerene cage hinders optimal device performance.^22^ Nevertheless, series of different isomers can be very good candidates to set up systematic studies of chemical structure-device behaviour relationships, since some of the molecular parameters of different isomers are similar to each other, while others are different. Previous studies of the effect of fullerene type on degradation used different fullerene materials containing different cage size, different side chains and different adduct numbers,^23^ making comparison difficult. In contrast, series of isomers of a single fullerene type allow us to conduct relatively well-controlled experiments. However, the traditional purification method of fullerene materials, high-performance liquid chromatography (HPLC), is not efficient for purifying materials containing numerous components.^24^ It is time-consuming to identify the molecular structure of each component, and this limits the amount of experimental work that can be done on isomer specific properties of higher adduct fullerenes. Our previous work demonstrated a multi-column peak-recycling HPLC method to purify the well-known bis-PCBM (dimethyl-4,4′-[3′*H*,3′′*H*-diphenyl-dicyclopropa(C_60_-I_h_)[5,6]fullerene-3′*H*,3′′*H*-diyl]dibutanoate) mixture. Bis-PCBM and its 19 isomers (except for chiral molecules) can be isolated, each with a purity of ∼99.9%.^25^ The molecular structures were also identified by the combined analysis of ^13^C NMR, UV-Vis absorption spectroscopy, and HPLC retention time analysis.^26^ This series of bis-PCBM isomers provides a materials pool for various fundamental studies, based on which we can control the molecule parameters such as energy level, strain, crystallinity and total energy by varying the side chain locations.

Two main pathways for the light-induced degradation of fullerenes in air have been identified previously: the first is through singlet oxygen generation *via* energy transfer from the triplet excited state of the fullerene (or the triplet of the donor, in the case of a blend film), leading to the chemical reaction of oxygen with the fullerene cage to form epoxide, diol or carbonyl defects on the cage, which can act as electron traps in the blend and affect the device performance.^4,5,27–31^ The second pathway is through superoxide (O_2_^−^) radical generation *via* photo-induced electron transfer from the fullerene to molecular oxygen followed by chemical degradation of the electron donor or acceptor upon reaction with O_2_^−^.^14,17^ It has been reported that the amount of O_2_^−^ formed is correlated to the LUMO level of the acceptor, such that a raised acceptor LUMO level leads to a greater O_2_^−^ yield and faster device degradation. However, it is not known how other molecular parameters affect these reactions nor to what extent the empirical observation that degradation rate correlates with LUMO is upheld in general, since the molecules used in the related study are diverse in multiple aspects.^17,32,33^ In the case of photodegradation in the absence of air, the device performance loss has been correlated to fullerene photo-dimerization, which can be suppressed by side-chain hindrance such as by using bis-PCBM instead of PCBM.^13,34^ However, it is not well known whether fullerene dimerization dominates the anaerobic degradation nor how other molecular properties (aside from the side-chain hindrance influence) affect this photodegradation rate. Molecular-level properties such as reaction energetics and cage strain can affect the thermodynamics and reactivity, respectively, which may also affect the material photostability.

In this work, we address the molecular property – degradation rate relationships for a series of purified bis-PCBM isomers. We determine their energy levels, structure and microstructure by several experimental and theoretical methods. Then, these isolated isomers are used to fabricate solar cell devices using identical recipes with the same-batch poly[*N*-9′-heptadecanyl-2,7-carbazole-*alt*-5,5-(4′,7′-di-2-thienyl-2′,1′,3′-benzothiadiazole)] (PCDTBT) as the donor polymer, chosen because this polymer is reported to have a high degree of photochemical stability.^35–37^ Since the PCDTBT:bis-PCBM blends are amorphous,^4^ the choice of this polymer should minimise problems of varying blend morphology. The obtained devices are then aged under one-sun light intensity either in ambient air or in N_2_ atmosphere, to study both the degradation in the presence or the absence of oxygen. The ability to isolate specific isomers enables a detailed analysis of the relationship between degradation and molecular parameters to a degree that is seldom present in previous studies. In our study we show that, once the impact of crystallinity is separated out, the previously suggested relationship between photodegradation in air and LUMO energy could be confirmed. We also propose a strategy to influence degradation *via* fullerene photodimerization *via* controlling the side chain positions and orientations.

## Results and discussion

### Chemical structures

**Fig. 1:**
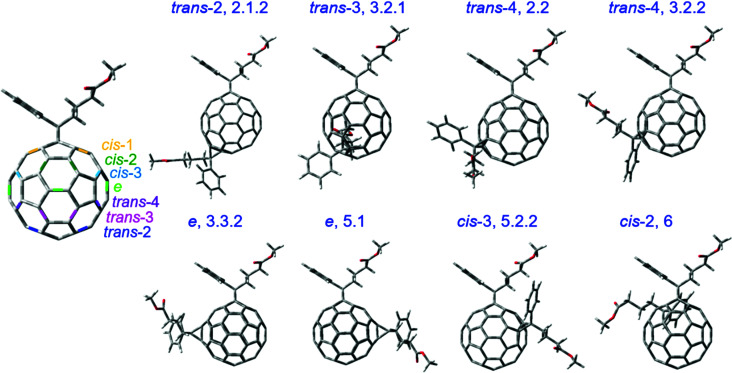
The molecular structures of the bis-PCBM isomers selected for degradation study. The *trans*, *cis* and *e* type positions on C_60_ cage are indicated with the first addend on the pole.

Eight selected isomers from six isomer groups are used in this study since other isomers tend to aggregate or form poor blends with PCDTBT. The eight isomers mainly differ in the position of the side chains as shown in Fig. 1. Isomers are named according to the HPLC fraction names as described in our previous work.^25,26^ Changing the position of the side chains will inevitably affect the properties of the molecule such as orbital energy levels, electron affinity, crystallinity, total energy, fullerene cage strain, and reactivity. Before quantifying such molecular properties theoretically, we note that the isomers show different redox properties when characterised using cyclic voltammetry (CV).^25^ The LUMO energy of an electron acceptor molecule is considered to influence its photodegradation significantly.^17^Table 1 shows that the LUMO energies, estimated from the reduction potentials measured by CV, vary by 100 meV across the series of isomers studied here with the isomer 2.1.2 showing the lowest value and isomer 3.2.2 showing the highest value. The measured LUMO values compare well across the isomer series with values calculated using density functional theory (DFT) except for the two *e* isomers (isomers 3.3.2 and 5.1), where the calculated LUMO values (the calculated HOMO energy plus the transition energy of the first excited state calculated using time-dependent DFT) overestimate the measured values by up to 0.2 eV (see Fig. S1a, ESI†). We then considered the crystallinity of the pristine isomer films (see Fig. S1b, ESI†) and found that isomers 2.1.2, 3.2.1, 2.2, 3.2.2, 3.3.2 and 5.1 showed no crystallization peak and can be considered amorphous whereas isomers 5.2.2 and 6 are relatively more crystalline. To compare the chemical structure of the molecules, we used the optimised geometry from DFT calculations in the ground state of the different isomers. The first carbon atoms of each of the two butyl chains (see Fig. S2, ESI†) on the fullerene cage varies with the chain location and orientation for different isomers while not varying with alkyl chain rotation. Herein, we use the distance between these two carbon atoms as an indicator of the molecular structure differences of different isomers. The value of the distance between these two carbon atoms for each bis-PCBM isomer measured from the geometry-optimised molecular structure is listed in Table 1, along with the calculated total energy of the optimised isomer stated relative to the total energy of isomer 6.

**Table 1:** Molecular parameter statistics of selected bis-PCBM isomers. Total energies are given relative to the total energy of optimised structure 6. LUMO energies are estimated from reduction potentials measured by CV. The values are identical to those reported for the same isomers in ref. 25

Isomer	Type	LUMO/eV	Shortest C=C bond/Å	Longest C=C bond/Å	Side-chain distance/Å	Total energy/meV	Largest epoxidation energy/eV
2.1.2	*trans*	−3.84	1.377	1.395	11.20	−132.52	1.61
3.2.1	*trans*	−3.78	1.377	1.394	10.08	−149.66	1.65
2.2	*trans*	−3.82	1.378	1.395	10.62	−119.46	1.64
3.2.2	*trans*	−3.73	1.378	1.395	9.61	−119.73	1.63
5.1	*e*	−3.79	1.377	1.394	7.45	−149.39	1.64
3.3.2	*e*	−3.79	1.378	1.394	9.17	−171.43	1.64
5.2.2	*cis*	−3.80	1.363	1.393	6.89	−72.93	1.68
6	*cis*	−3.82	1.358	1.395	6.29	0	1.71

### Theoretical assessment of reactivity

The reactivity of a fullerene can be related to the degree of, and spatial variation in, the strain on the C_60_ cage that is induced by curvature. Strain can be represented by the pyramidalization angle of the carbon atoms.^38,39^ For uniformly curved fullerenes or nanotubes, higher pyramidalization angle indicates greater strain,^40^ and is likely to indicate lower molecular stability or greater reactivity. In the case of the fullerene adducts, adduction changes the strain locally and since the fullerene cage usually breaks from the weakest bond, the difference in the bond length in a bis-isomer relative to the average bond length C_60_ can represent the relative degree of strain for bis-PCBM molecules. Taking the C=C bond length of C_60_ as the original state, we extract the longest (stretched strain) and shortest (compressed strain) bond lengths among the 28 C=C bonds for each bis-PCBM isomer following molecular structure optimisation. We list the results in Table 1.

A well-established degradation pathway for fullerenes exposed to light and air is the epoxidation of the fullerene cage by oxygen attachment to a C=C bond followed by diol or carbonyl formation (as shown in Fig. S3, ESI†). This process is believed to follow the formation of singlet oxygen by energy transfer from a triplet state on the fullerene or another molecule. We evaluate the likelihood of this chemical reaction first by evaluating the triplet energies of all fullerene isomers using time-dependent DFT (Fig. S1c, ESI†), and then by evaluating the energy released by epoxidation of the different isomers.^5^ Taking PCBM–O (Fig. S2, ESI†) as an example, we calculated the total energy of all potential PCBM epoxide molecules. It is clear from the energy of epoxidation for different C=C bonds in Table S1 (ESI†) that oxygen tends to occupy the C=C bond near the side chain, *i.e.* the *cis*-1 positions. Further analysis of the bond length shows that *cis*-1 bonds have the shortest bond lengths and will experience compressive bond strain (taking the C=C of C_60_ as reference) compared to other types of C=C bonds with enlarged bond lengths or less compressed bond lengths. Similarly, for bis-PCBM isomers, the eight C=C bonds that are near to the two side chains, like the *cis*-1 bonds for PCBM, are also the shortest eight bonds in each of the isomers after molecular structure optimisation. This finding indicates that epoxides are most likely to form on these C=C bonds around the side chains when the material is exposed to light and air. To compare the relative tendency to oxidise of bis-PCBM isomers, the total energy of the eight most likely bis-PCBM epoxides for each isomer were all calculated as listed in Table S2 (ESI†), where the energy Δ*E* released during oxidation was calculated using the formula: Δ*E* = [*E*_T_(isomer) + *E*_T_(O_2_)/2] − *E*_T_(epoxide) where *E*_T_(*X*) refers to the total energy for component *X* calculated using DFT.^5^

### Optical photodegradation of blend films in air

**Fig. 2:**
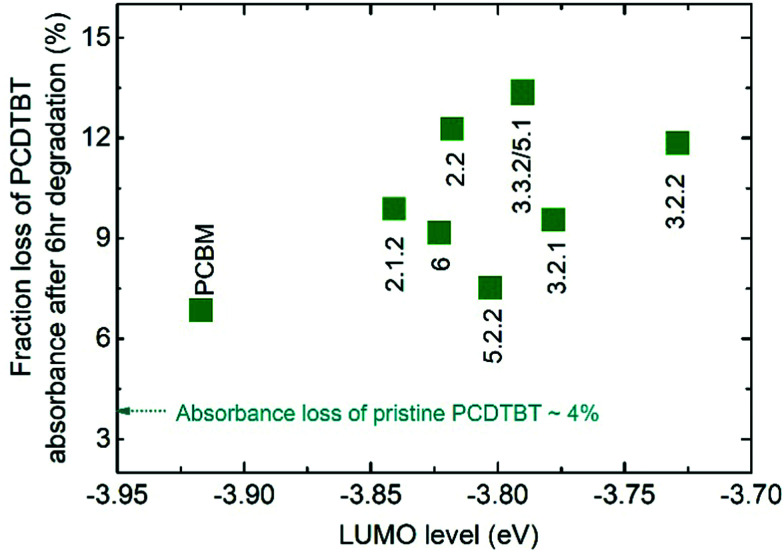
Fraction of initial polymer absorbance at 571 nm that is lost after 6 h exposure to AM 1.5G illumination in air for each PCDTBT:fullerene blend films as a function of the measured LUMO level of the acceptor. Also indicated is the absorbance loss of the pure polymer film. The absorbance measurements of neat fullerene films are not shown since there was no significant change after 6 h illumination, in agreement with results in the literature.^30^

We first assess the effect of photodegradation on the optical properties of both the pristine fullerene films and blend films of the fullerene with PCDTBT. All the films were exposed to simulated AM 1.5G illumination in air and their absorbance was measured after 1, 3 and 6 hours. Whilst the absorbance of the pristine fullerene films did not change after 6 hours of exposure, the absorbance of the PCDTBT:fullerene blend films reduced by up to 13% around the peak of the polymer absorption. This loss in the polymer absorbance can be explained by the formation of superoxide *via* electron transfer from either fullerene or polymer to oxygen. A shallow LUMO level could facilitate the transfer of electrons to molecular oxygen and increase the yield of superoxide, which in turn reacts with the polymer leading to a bleaching of the polymer absorbance.^17,33^Fig. 2 shows the fraction of initial polymer absorbance, at the PCDTBT absorption peak of 571 nm, that is lost after 6 h of exposure to AM 1.5G in air for each of the blend films. The absorbance loss data are plotted as a function of the measured LUMO level of the fullerenes along with the absorbance loss of a pristine PCDTBT film aged under the same conditions. All blend films show a higher polymer absorbance loss than the pristine polymer (4.5%), with PCBM-blend showing a 6.8% loss and isomer-5.1-blend showing the highest loss of 13.4%. It is thus clear that both PCBM and bis-PCBM isomers accelerate the polymer photobleaching. Whilst the absorbance loss is generally higher for the higher-lying LUMO bis-adducts than for PCBM, there is no clear correlation of PCDTBT absorbance loss with fullerene LUMO level in contrast to the behaviour reported by Speller *et al*.^17^ Nevertheless the generally higher losses for blends with bis-adducts, which have higher lying LUMO energies than PCBM, is consistent with easier superoxide formation for those adducts than for PCBM during photodegradation of the PCDTBT:fullerene blends in air.

### Photodegradation of blend devices in air

**Fig. 3:**
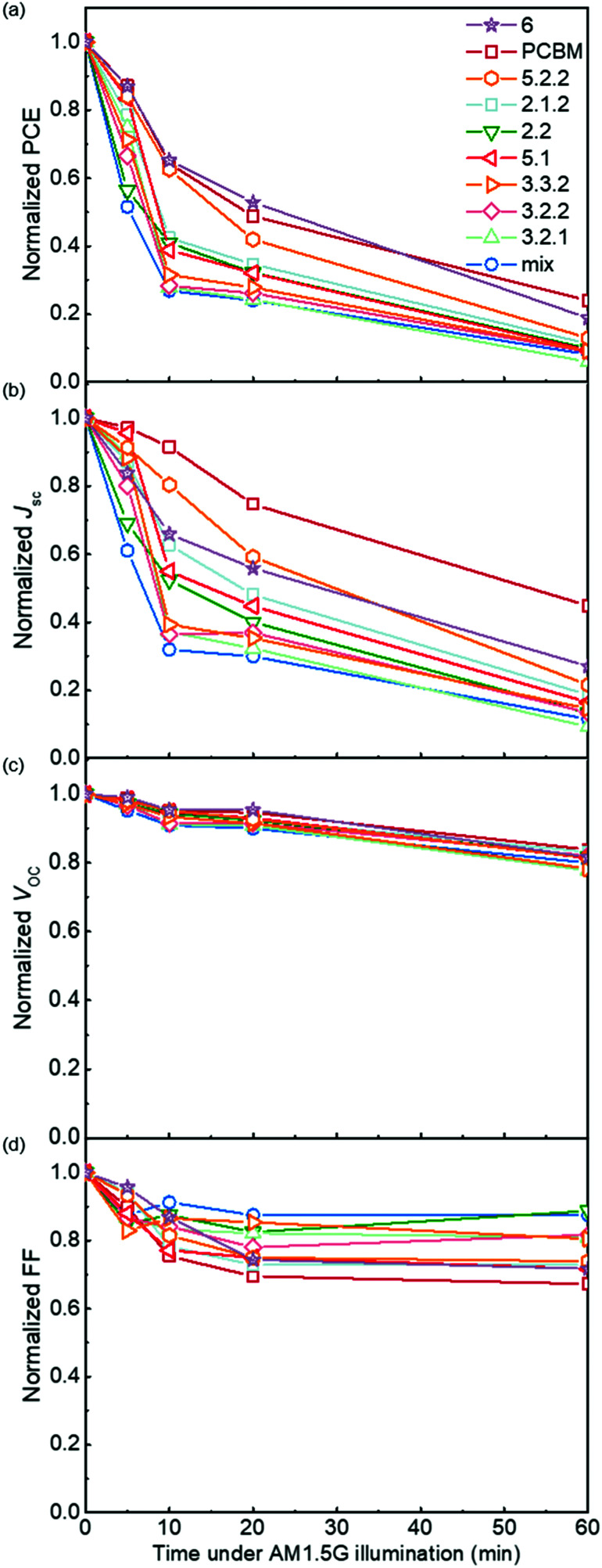
Photovoltaic parameters (a) power conversion efficiency (PCE), (b) short circuit current density *J*_sc_, (c) open circuit voltage *V*_oc_ and (d) fill factor FF of PCDTBT:fullerene devices as a function of the time for which the active layer had been exposed to simulated AM 1.5G illumination in air prior to electrode deposition. All performace parameters are normalised to the value measured for a device whose active layer was not exposed to air.

Following the optical degradation of the films in air, we proceed to investigate the degradation of solar cell devices after exposure of the active layer to light in air. In order to reduce the impact of the photodegradation of the interlayers and the electrodes on the degradation of the devices, unfinished devices consisting of substrate, bottom interlayer and active layers were prepared in an N_2_ filled glovebox, then exposed to simulated AM 1.5G illumination in air for different periods of time before being transferred back to the glovebox for the deposition of the interlayer and top electrode. The evolution of performance parameters as a function of exposure time to simulated AM 1.5G illumination in air is summarised in Fig. 3 for blend devices with the structure glass/ITO/PEDOT:PSS/PCDTBT:fullenere/PFN/Al. All values were normalised to their respective initial values before degradation. The un-normalised *J*–*V* characteristics are shown in Table S3 (ESI†) with typical *J*–*V* curves before and after degradation shown in Fig. S4a (ESI†). The bis-PCBM isomer-based devices show a range of degradation rates. The *cis* isomers 6 and 5.2.2 show the lowest PCE loss, similar to that of the PCBM devices, while the PCEs of the other isomers drop faster, with the bis-mixture showing the worst performance. It is clear that the devices degrade faster than what could be inferred from the blend film absorbance degradation under the same exposure conditions. This indicates that the photo-bleaching induced absorption loss is insufficient to explain the device performance loss, and suggests that the device performance loss must also arise from electrical degradation such as lower charge-carrier mobility resulting from defects formed during photo-oxidation of the active layer.^4,41^ We note that the degradation observed here is faster than what would be observed in finished devices because of the lack of interlayer and top electrode that would normally suppress the oxygen permeation and decrease the active layer oxidation rate. In this study, we wished to distinguish isomer-specific active layer sensitivity from any reactions of top electrode metal (aluminium) to oxygen and water that could obscure the effect of the isomer degradation. We stress that for practical applications, encapsulation is currently the best method to prevent device photodegradation in air.^42^

**Fig. 4:**
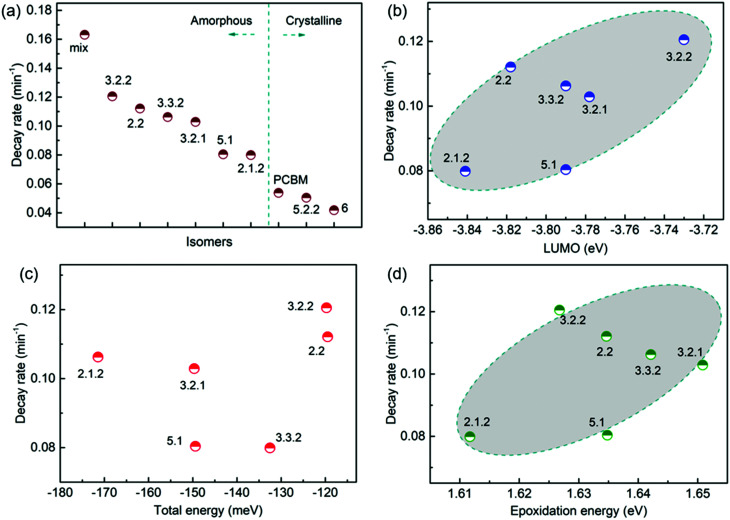
Correlation between the PCE decay rate and molecular parameters: (a) crystallinity (note that the order of isomers within each group, amorphous and crystalline, is not significant), (b) LUMO energy estimated from CV measurements, (c) calculated total energy, (d) largest calculated epoxidation energy.

To evaluate how particular molecular parameters affect the fullerene degradation, the performances of devices made from crystalline or amorphous isomers were compared in separate groups. We looked for correlations between the PCE decay rate, characterised by fitting an exponential decay to the data (see Fig. S4b, ESI†), and, in turn, the total energy of the isomer, its LUMO energy, the energy of its first triplet excited state, the energy of epoxide formation and the crystallinity of the isomer. Fig. 4a compares the degradation behaviour of blend devices of the two more crystalline isomers, 5.2.2 and 6, and crystalline PCBM with that of the more amorphous isomers, 2.1.2, 2.2, 3.2.1, 3.2.2, 3.3.2 and 5.1. The higher PCE decay rate for the amorphous compared to the crystalline group indicates a lower tendency for the crystalline isomers to take part in the oxidation reaction, which may be either epoxidation of the fullerene or superoxide formation. A likely reason is that denser fullerene packing inhibits the permeation of oxygen through the material and inhibits the reaction of oxygen with fullerene.^18,19^ The results are also consistent with the previous report that films containing less aggregated PCBM are more susceptible to oxidation in air.^30^ Also shown, for the six amorphous isomers, are the PCE decay rates as a function of isomer LUMO energy measured by CV (Fig. 4b), calculated isomer total energy (Fig. 4c) and calculated energy of epoxidation (Fig. 4d). The most noticeable correlation is between the PCE decay rate and the isomer LUMO energy measured by CV (Fig. 4b). The rate of degradation tends to increase with higher lying LUMO energy. This trend also applies to the two crystalline isomers, since isomer 6, with a deeper LUMO level (−3.82 eV), is more stable than crystalline isomer 5.2.2 (LUMO −3.80 eV). This correlation between stability and depth of LUMO energy is consistent with the superoxide degradation mechanism, whereby a deeper LUMO slows down the rate of electron transfer from fullerene to oxygen to form O_2_^−^. The degradation rate of amorphous isomers shows no correlation with total energy as indicated in Fig. 4c. We then analysed the correlation between degradation rate and the energy released during epoxidation for the amorphous isomers (see Fig. 4d), which is unclear but may indicate a positive correlation. We further analysed the correlation between PCE fractional loss and the above molecular parameters (see Fig. S5, ESI†) at different degradation time, which also exhibited obvious correlation with the crystallinity and LUMO level and negligible correlation with either the total energy, the energy released upon first epoxidation, or the energy released in subsequent oxidations. Comparison of fullerene isomer triplet energy with degradation rate showed no clear correlation (Fig. S6, ESI†), suggesting that faster degradation is not caused by more energetic triplet states.

Although crystalline isomers degrade slower than amorphous ones, the degree of crystallinity does not dominate the degradation absolutely, since isomer 5.2.2 (with higher crystallinity) shows faster degradation than isomer 6 (with less crystallinity) as indicated in Fig. S7 (ESI†). Since isomer 6 has a higher LUMO and a higher epoxidation energy than isomer 5.2.2, it is possible that even among the crystalline isomers the LUMO and epoxidation energy play a role. In addition, when isomers have similar LUMO level and crystallinity, the ones with lower total energy show less tendency to oxidize and are more stable (see details in ESI,† Fig. S7). Overall, the photodegradation of bis-PCBM based organic solar cells in air is most clearly correlated to the LUMO energy and to the molecular crystallinity. We assign these correlations to the effect of LUMO energy on superoxide formation and the effect of molecular packing on diffusion of molecular oxygen, respectively. Although the epoxidation energy didn’t show clear correlation with the PCE loss (Fig. 4d), our data suggest there may be some relationship between degradation rate and the driving energy for epoxide formation, that could warrant further study. However, there was no clear evidence that fullerene degradation is dominated by the precursor to the fullerene epoxidation, which is believed to be singlet oxygen generation mediated by triplets.^30^ Since the triplet energies of bis-PCBM isomers are similar and show no clear correlation with the PCE decay rate (see ESI,† Fig. S6), differences in the driving energy for singlet oxygen formation should not be responsible for the variation of the PCDTBT:bis-PCBM degradation, even if differences in epoxide formation probability are relevant. Considering also the observation of enhanced polymer absorbance loss for the bis-adducts, the data presented here suggest that the photodegradation of PCDTBT:bis-PCBM blends is more likely to be dominated by the superoxide formation mechanism than by the pathway involving triplet-mediated singlet oxygen generation.

### Anaerobic degradation of blend devices

**Fig. 5:**
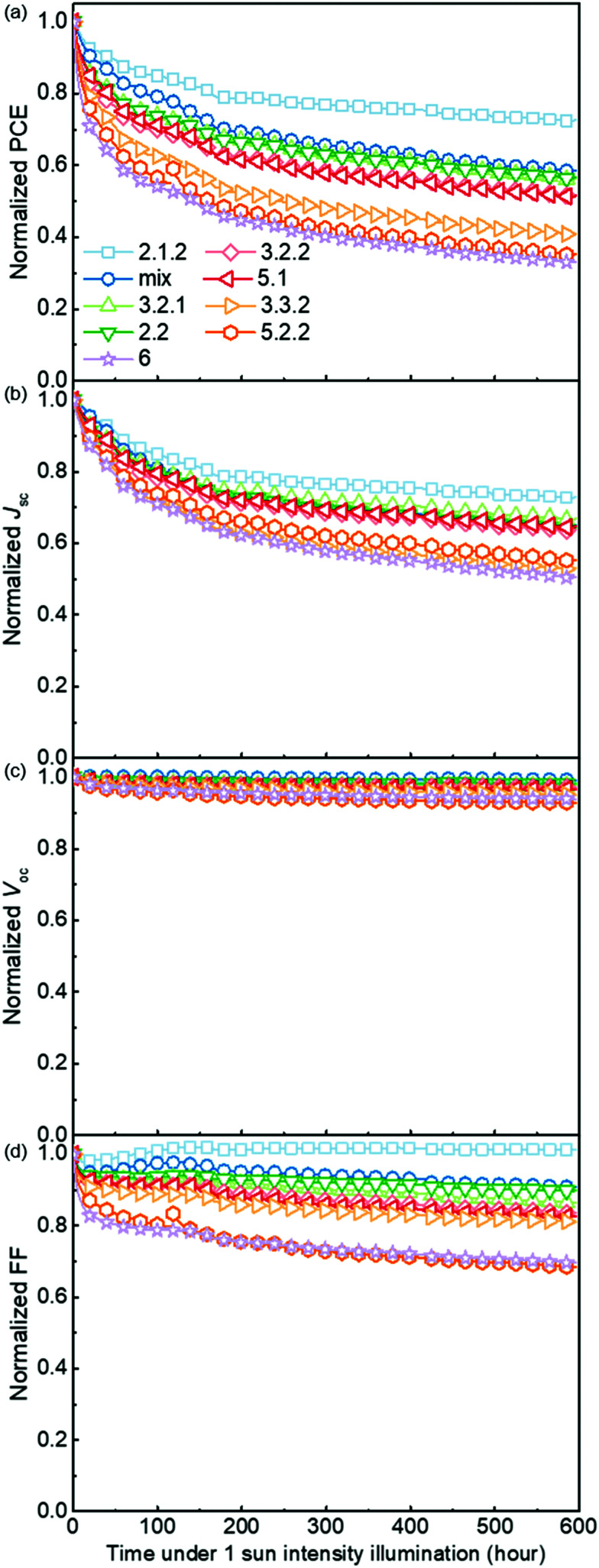
Evolution of normalised device parameters of the PCDTBT:bis-PCBM devices under continuous 1 sun intensity illumination in a nitrogen atmosphere. All parameters are normalised to their initial performance.

Fig. 5 demonstrates the evolution of device parameters under continuous illumination in N_2_. Illumination equivalent in intensity to one sun were provided by a white light LED array and devices were held at open circuit condition between measurements. All parameters are normalised to their initial performance. The *J*–*V* characteristics are shown in Fig. S8 (ESI†). Both the PCE and *J*_SC_ of the bis-PCBM isomer-based devices decrease following an approximately exponential decay with time while *V*_OC_ and FF do not fall significantly except for the FF of isomers 5.2.2 and 6. Therefore, the observed PCE loss is dominated by the loss of *J*_SC_. The rates of anaerobic photodegradation of different isomers differ considerably, with the PCE at 600 h varying from 35% (isomer 6) to 80% (isomer 2.1.2) of its initial value. In contrast to what was observed under aerobic conditions, the bis-PCBM mixture-based device displays relatively stable performance, with PCE remaining at 60% after 600 h, which is higher than most isomer-based devices. The improved stability of the bis-PCBM mixture under anaerobic conditions may be expected since anaerobic photodegradation has previously been assigned principally to fullerene dimerization,^15^ and dimerization of the bis-PCBM mixture is likely to be hindered by the varying side chain positions of the isomers in the mix. For a more detailed comparison, we fit the PCE decay rate to a single exponential decay for each isomer blend (see Fig. S4c, ESI†) and compare these decay rates to different molecular parameters of bis-PCBM isomers.

**Fig. 6:**
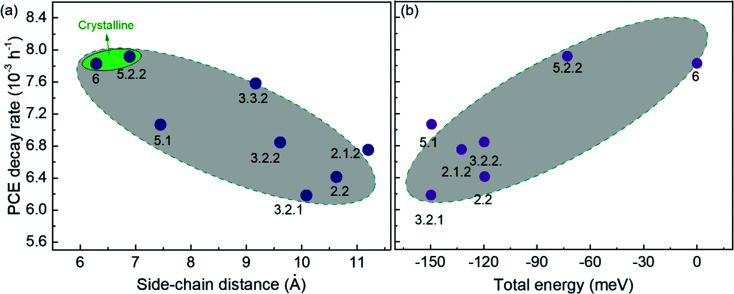
Comparison between PCE decay rate and the fullerene molecular parameters of the PCDTBT:bis-PCBM devices degraded anaerobically: (a) crystallinity and side-chain distance; (b) total energy.

It has been shown that C_60_ and PCBM can dimerise by forming covalent intermolecular C–C bonds between two adjacent fullerene molecules through a ‘2+2’ cycloaddition reaction under the irradiation of visible or ultraviolet light.^16,43,44^ The reaction occurs between two parallel double bonds on adjacent fullerene cages and can be hindered by the presence of side chains. Fig. 6a shows the PCE decay rate as a function of the distance between the two side chains on the fullerene cage, here called the ‘side-chain distance’. The data show a clear positive correlation relationship such that the greater the separation of the side chains, the greater the degradation rate. This observation is consistent with the dimerization mechanism of degradation where two fullerene cages need to approach each other to within a few Angstroms. Fig. 6a also shows that the isomers with shortest side-chain distances, and faster anaerobic photodegradation, are crystalline while the other ones are amorphous. The faster degradation of the more crystalline isomers suggests that condensed packing of molecules can promote dimer formation. Fig. 6b shows the comparison between the PCE decay rate and total energy of different isomers, indicating an overall trend whereby the more thermodynamically stable isomers are also more stable with respect to anaerobic photodegradation. The comparison between the PCE loss at different times (rather than the decay rate) and the side-chain distance and isomer total energy (Fig. S9, ESI†) show similar correlations, although the total energy appears to have an inferior influence to side-chain separation. To distinguish the effects of side-chain distance and total energy on degradation, the isomers were further separated into two groups, *i.e.* with large side-chain distance (∼10.5 Å) and short side-chain distance (∼7 Å). As displayed in Fig. S9c (ESI†), while the large side-chain distance group shows no obvious correlation of total energy with degradation, the isomers in the short side-chain distance group show a positive correlation of total energy with degradation rate. Therefore, the lower stability of isomers with higher total energy may also facilitate device degradation. The device degradation shows no clear correlation with the molecular LUMO level as indicated in Fig. S10 (ESI†).

Overall, the anaerobic photodegradation of bis-PCBM isomer-based devices is consistent with fullerene cage dimerization being the dominant mechanism.^13,15,45^ Crystalline isomers have close molecular packing which can facilitate dimer formation, while those with close side chains also present larger cage surface area for close contact with other fullerenes. Meanwhile, the thermodynamic stability of the isomers (inferred from total energy) may influence the degradation to a secondary degree.

## Discussion

Herein, based on blend devices made from PCDTBT and separated isomers of bis-PCBM, we have investigated the correlations between device photodegradation in the presence or absence of air and different molecular parameters that depend on the positions of the two side-chains on the C_60_ cage. The results show that the photo-degradation of PCDTBT:bis-PCBM devices in air is consistent with superoxide formation being a major degradation pathway, with the degradation rate strongly correlated with the LUMO energy level of the isomers. The pathway involving epoxide formation following triplet-mediated singlet oxygen formation in PCDTBT:bis-PCBM blends is also expected to occur, but we found a weaker correlation between photodegradation rate in air and the likelihood of epoxide formation, which we inferred from the calculated energy of epoxidation, and no correlation between photodegradation rate and fullerene triplet energy. The superoxide pathway is also consistent with the observed photo-bleaching of the polymer and with the even greater loss of photocurrent, suggesting the formation of electronic defects following oxidation. It is noted (Fig. 4a) that more amorphous isomers tend to result in a higher device decay rate, consistent with previous studies. Whilst this is consistent with crystallinity inhibiting oxygen access to the bulk of the film, it doesn’t distinguish between the two degradation pathways.

The rate of anaerobic photodegradation in N_2_, sometimes known as burn-in degradation, of PCDTBT:bis-PCBM devices exhibits a clear negative correlation with the separation of the two side-chains of bis-PCBM, suggesting that the degradation may be dominated by fullerene cage dimerization, since short side-chain distance means greater opportunity to form a dimer. Crystalline isomers also have close molecular packing which may facilitate dimer formation. The isomers with higher total energy are less stable and this may enhance the anaerobic photodegradation, but this plays inferior role in the overall device degradation. The LUMO level shows no correlation with anaerobic photodegradation.

Although fullerenes may take part in different degradation pathways when blended with different donor materials, our findings can be expanded to provide general guidelines from the perspective of fullerene molecular design. For example, to eliminate the material's oxidation in air, crystallinity should be improved; to cut down on the oxidation pathway of materials degradation, the LUMO energy should be deep. To prevent epoxidation, epoxidation energy should be constrained to a low value. For the findings from anaerobic photodegradation, dimerization can be controlled *via* changing the side chains. The molecular total energy should also be deep to maximise thermodynamic stability. All of these parameters can be controlled, in principle, through design of chemical structure.

As with fullerenes, the position of the LUMO level and the film crystallinity have emerged as critical parameters in the design of efficient NFAs. For example, the current most dominant NFAs are primarily based on the ITIC, IDTBR and Y6 families, which are optimised *via* the arrangement of Donor (D) and Acceptor (A) components as well as side chains, with molecular energy level, morphology and crystallinity used as critical indicators.^3,46–50^ Therefore this study's conclusions on the relationship between crystallinity, epoxidation or total energy and the photostability of fullerenes may also be applicable to the design of photostable NFAs.

Further, our work demonstrates an approach wherein chemically similar molecular structures can be used to investigate how molecular parameters affect degradation and where the impact of different molecular properties on degradation mechanisms can be separated. A similar method could be used to study sets of NFAs with similar chemical structures to both validate the findings of this work and seek further correlation between molecular properties and degradation pathways. Such studies will help to bridge the efficiency-stability gap of high performing organic solar cells.

## Conclusions

In conclusion, organic solar cell devices based on isolated bis-PCBM isomers and PCDTBT were degraded under 1 sun illumination in air and in nitrogen. The degradation behaviour of the selected bis-PCBM isomer devices was compared with the isomers' molecular parameters to extract correlations. This work suggests that photodegradation can be regulated at the molecular level. For example, the LUMO level should be controlled to lie at a low level, the epoxidation energy should be low and the material crystallinity should be improved to slow down photodegradation in air. For anaerobic stability, the total-energy related thermodynamics have greater correlation to the blend photodegradation, while the crystallinity and molecular structure should be well controlled to modulate the device morphology. The work provides new insights and a methodology to investigate the effect of molecular parameters on device degradation. These findings not only complement the current degradation mechanism of fullerene materials and further promote the application of higher adduct fullerenes, but also provide a reference for the future investigation on environmentally stable materials.

These results demonstrate the value of studying the relationship between properties of isolated isomers and degradation of devices based on those isomers. We suggest the approach could be extended to other molecular property-device performance relationships.

## Experimental

### Materials

The synthesis of bis-PCBM followed the classic procedure to produce PCBM derivates, which was reported by Hummelen *et al.*^51^ The raw material, their quantities, and the reaction conditions were based on the detailed method in Dr Ricardo Bouwer's PhD thesis, which was supervised by Prof. Hummelen.^52^ All reagents and solvents were used as received from Sigma Aldrich. Then the as-synthesized bis-PCBM mixtures were purified by the multi-column peak-recycling HPLC method as demonstrated in our previous work.^25^ Each of the selected isomers was isolated with each of purity of ∼99.9% and were condensed to supersaturated solution and then was transferred into a vacuum for slow drying for three days. PCDTBT was purchased from 1-material. The aqueous dispersion of poly(3,4-ethylenedioxy-thiophene):poly(styrenesulfonate) (PEDOT:PSS) (Clevios PVP AI 4083) was purchased from H. C. Starck Inc. The electron transport layer poly(9,9-bis(3′-(*N*,*N*-dimethylamino)propyl)-2,7-fluorene)-*alt*-2,7-(9,9-dioctyl fluorene) (PFN) was dissolved in methanol with addition of 0.25 vol% acetic acid with a concentration of 0.2 mg ml^−1^ solution. All purchased materials and solvents were used as received without further treatment unless otherwise noted.

### Device fabrication

The device structure in this study is ITO/PEDOT:PSS (30 nm)/PCDTBT : fullerene (1 : 2, w/w) (80 nm)/PFN (5 nm)/Al (100 nm). The PEDOT:PSS anode buffer layer was spin-cast on the precleaned ITO anode substrate then dried in air at 150 °C for 15 min. Then, a thin layer of PCDTBT:fullerene was deposited by spin coating in chlorobenzene solution, and solvent annealing treatment for 90 s in a Petri dish containing 2–3 mL of tetrahydrofuran. Soon after the treatment, a 5 nm PFN layer was spin-coated from methanol solution onto the active layer. Subsequently, the films were transferred to air for photo–air degradation or to an evaporator for Al cathode deposition. The top electrode evaporation was carried out at a rate of 0.1 Å s^−1^ through a shadow mask with an aperture area of 0.045 cm^2^ under a constant pressure of less than 10^−6^ torr.

### Photodegradation in air

To study photodegradation of the active layers in air, active layers (thickness 80 to 90 nm) were spin-coated on to PEDOT:PSS coated ITO substrates inside an N_2_ filled glovebox. After transferring out from the glovebox, the active layers were placed in a sample holder in air (relative humidity ∼40%) under a xenon lamp with AM 1.5G filters and subject to illumination by a 100 mW cm^−2^ solar simulator (Oriel Instruments) for different periods of time. The unfinished devices were transferred back into the glovebox for interlayer and top electrode (PFN/Al) evaporation after 5 min, 10 min, 20 min, or 60 min. The *J*–*V* characteristics of the series of devices with different exposure history were measured using a Keithley 2400 Source measure unit. The illumination intensity was adjusted with a calibrated KG-5 silicon diode from Newport.

### Anaerobic photodegradation

The cells were placed into an environmental chamber which was purged with a constant flow of nitrogen. A white LED array was used to illuminate the cells. The light intensity was set such that the *J*_SC_ approximately matched that measured under 1 sun AM 1.5G conditions. Devices were held at *V*_OC_ for the duration of the test and a *J*–*V* sweep was performed once per hour to monitor the performance. A water cooling system ensured that the environment temperature stayed below 26 °C throughout the stability test.

### Theoretical calculations

Geometry optimisation and electronic structural calculations for the bis-PCBM isomers and PCBM were performed using density functional theory (DFT) at the B3LYP/6-311G (2df, 2pd) level. The LUMO level was derived by adding the HOMO energy to the energy of the first singlet excitation calculated by time-dependent density functional theory (TD-DFT) using the same functional and basis set. Triplet energies were also calculated using TD-DFT. The calculations were carried out with Gaussian 09 software package under vacuum.^53^

### Measurements

The MALDI-TOF measurements were conducted in negative-reflection mode using an ultrofleXtreme mass spectrometer (Bruker Daltonics, Bremen, Germany), which is equipped with a Smartbeam-II Nd:YAG laser (*λ* = 355 nm). The samples were prepared by spin coating the isomer solutions onto clean ITO substrates. The DSC measurements were carried out with a TA instrument calorimeter (mode DSC2A-00503). Approximately 5 mg of sample was weighed into an aluminum pan and the pan sealed by pressing. The samples were left to equilibrate at 25 °C for 20 min then heated under nitrogen at a rate of 10 °C min^−1^ to 300 °C, held at this temperature for 5 min, then cooled at a rate of −10 °C min^−1^ to 25 °C.

## Conflicts of interest

The authors declare no competing financial interests.

## Supplementary Material

TC-010-D1TC05768E-s001
