# Supplementary material for: Relationship between molecular properties and degradation mechanisms of organic solar cells based on bis-adducts of phenyl-C61 butyric acid methyl ester
Source: J Mater Chem C Mater. 2022 Apr 25;10(20):7875–85. doi: 10.1039/d1tc05768e (PMC9134990; doi:10.1039/d1tc05768e)
Supplement: TC-010-D1TC05768E-s001 [file TC-010-D1TC05768E-s001.pdf]

Electronic Supplementary Material (ESI) for Journal of Materials Chemistry C.
This journal is © The Royal Society of Chemistry 2022

Supplementary Information of

Relationship between molecular properties and degradation mechanisms of organic solar cells based on bis-adducts of phenyl-C_{61} butyric acid methyl ester

Xueyan Hou,[a,b] Andrew J. Clarke,[c] Mohammed Azzouzi,[b] Jun Yan,[b] Flurin Eisner,[b] Xingyuan Shi,[b] Mark F. Wyatt,[d] T. John S. Dennis,[e,f] Zhe Li,[g*] Jenny Nelson[b*]

a International Collaborative Laboratory of 2D Materials for Optoelectronics Science and Technology of Ministry of Education, Institute of Microscale Optoelectronics, Shenzhen University, Shenzhen, 518060, China.

b Department of Physics and Centre for Plastic Electronics, Imperial College London, London, SW7 2AZ, United Kingdom.

c SPECIFIC, Swansea University Bay Campus, Swansea, Wales, SA1 8EN, United Kingdom.

d National Mass Spectrometry Facility, Swansea University Medical School, Singleton Park, Swansea, SA2 8PP, United Kingdom.

e State Key Laboratory of Motor Vehicle Biofuel Technology, International Research Center for X Polymers, Department of Polymer Science and Engineering, Zhejiang University, Hangzhou 310027, China.

f Haina-Carbon Nanostructure Research Center, Yangtze Delta Region Institute of Tsinghua University, Jiaxing, 314006, China.

g School of Engineering and Materials Sciences, Queen Mary University of London, London, E1 4NS, United Kingdom.

Corresponding authors: zhe.li@qmul.ac.uk, jenny.nelson@imperial.ac.uk

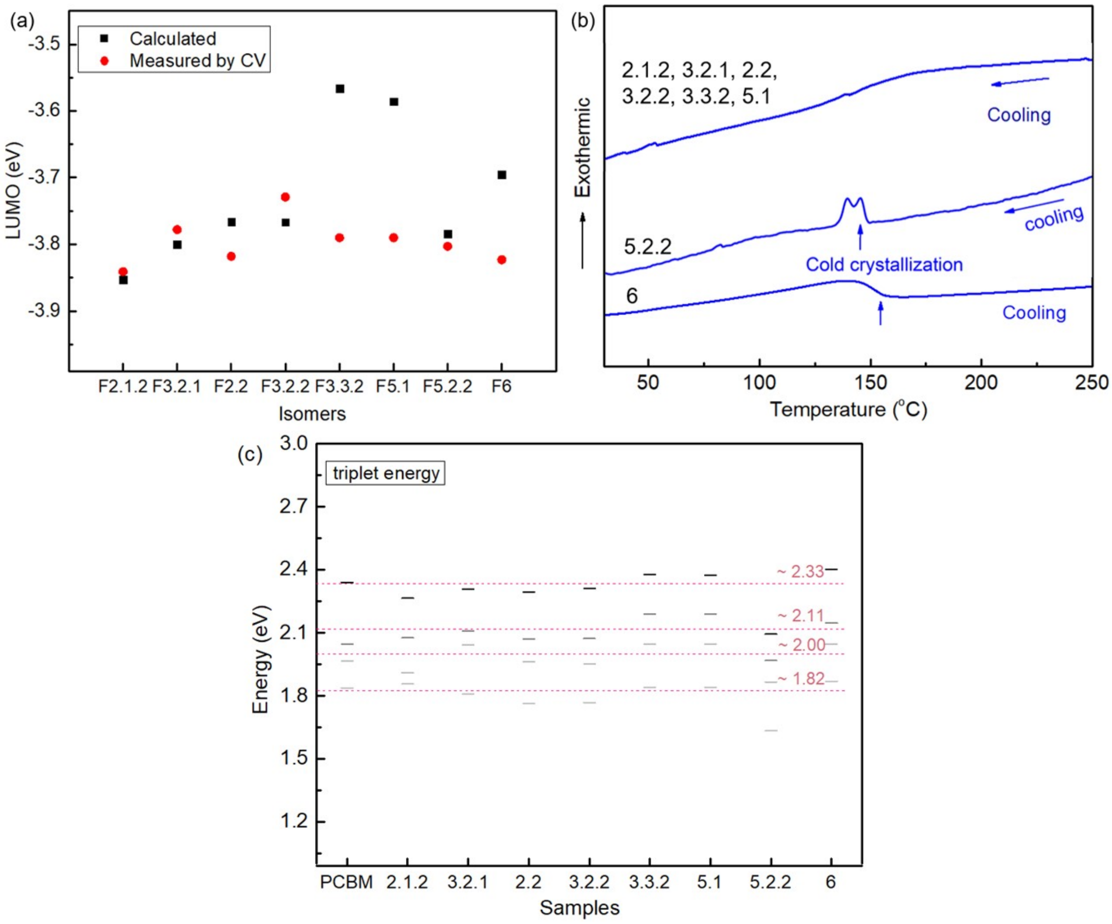

Fig. S1 (a) The isomer LUMO level statistics from Gaussian calculation and CV measurement. (b) Cooling process of the DSC test for different isomers. Isomers 2.1.2, 3.2.1, 2.2, 3.2.2, 3.3.2 and 5.1 show no crystallization peak and should be amorphous. Isomer 5.2.2 and 6 are relatively crystalline. (c) The calculated triplet energies (triplet state 1 to triplet state 4) of the isomers.

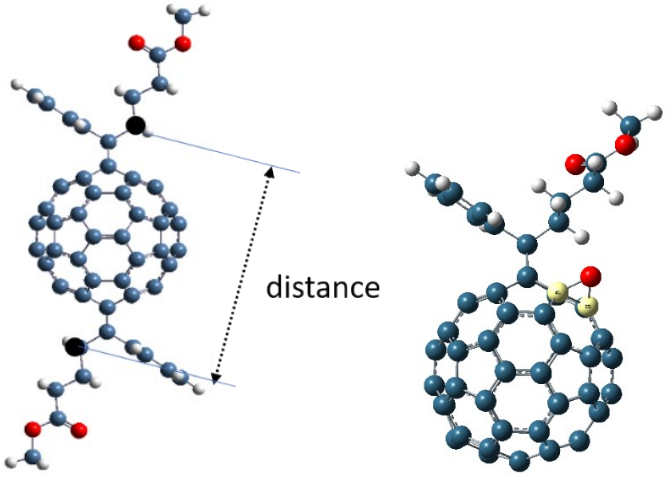

Fig. S2 Left: Illustration of side chain distance for bis-PCBM isomers. The side chain distance is used to represent the molecular structure difference and tendency to form dimer. Right: Epoxide with one oxygen near the alkyl chain (*cis*-1 position).

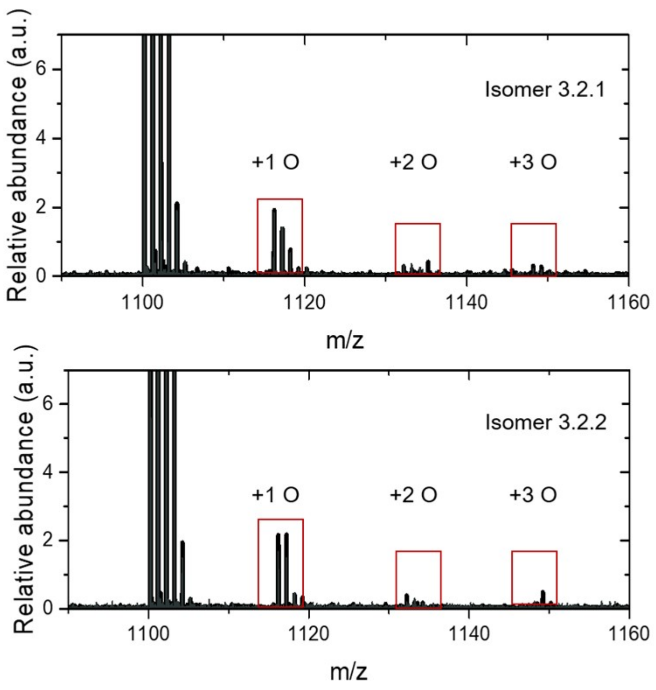

Fig. S3 MALDI-TOF measurement of bis-PCBM isomers 3.2.1 and 3.2.2 degraded in films under one sun in air for 60 mins.

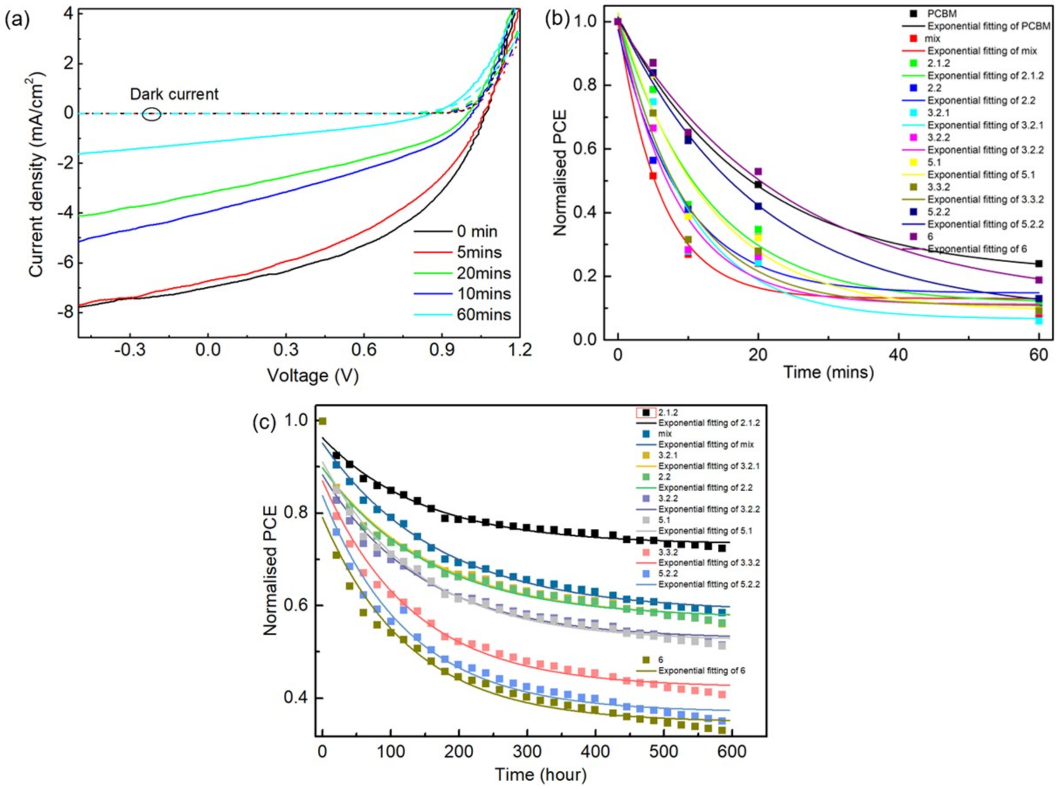

Fig. S4 (a) The typical *J-V* curves of isomer 5.1 before and after photodegradation in air for different time. (b) The exponential fitting of degradation performance under light and air. (c) The exponential fitting of degradation performance under light and N_2.

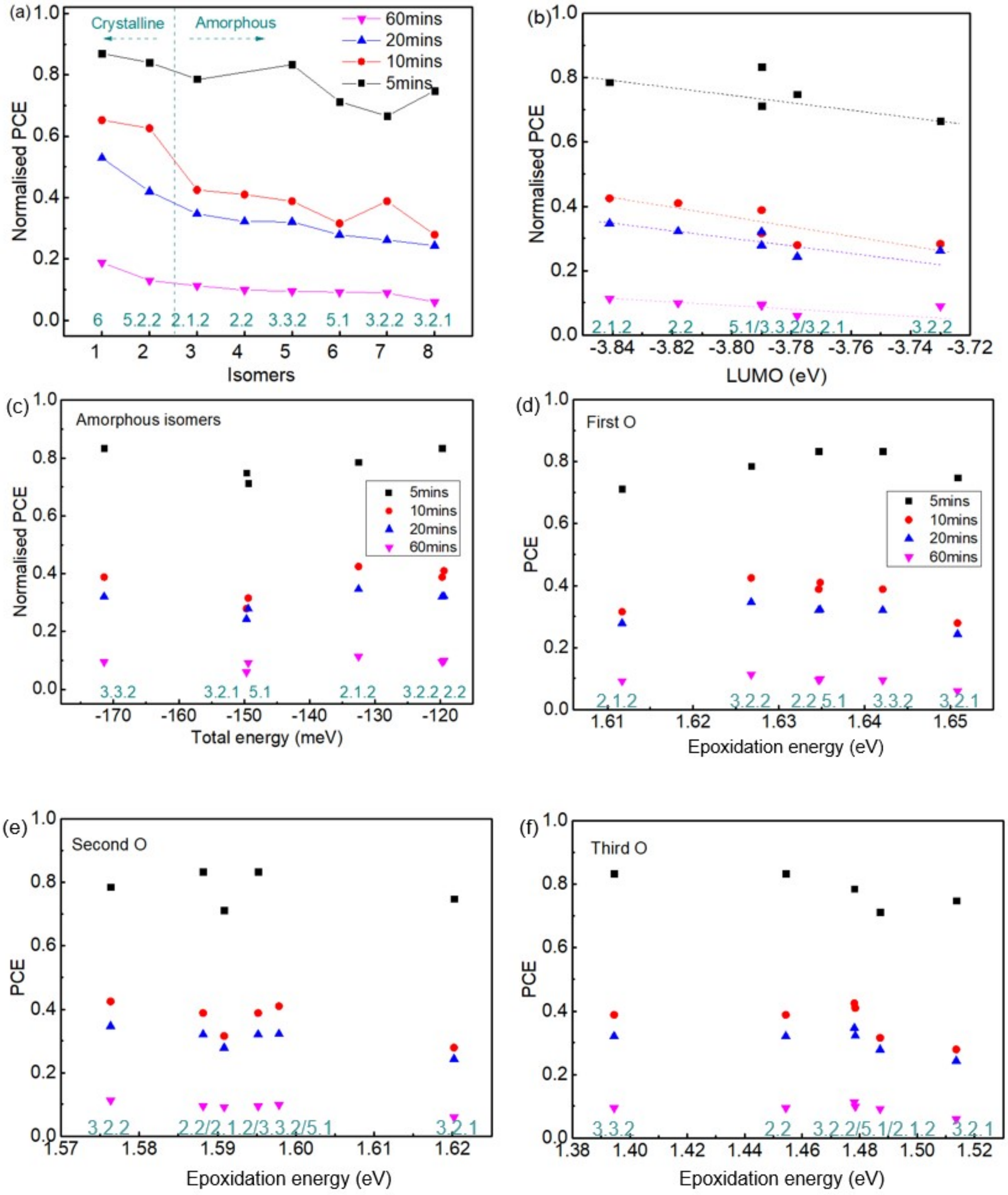

Fig. S5 Correlation between PCE loss and molecular parameters: (a) Crystallinity, (b) LUMO level, (c) total energy, (d-f) epoxidation energy.

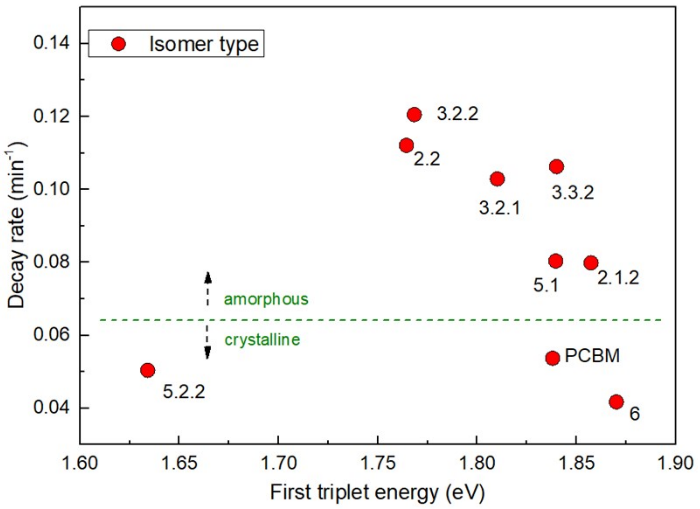

Fig. S6 Photodegradation rate of blend devices made with different fullerene isomers, plotted against calculated energy of the first triplet state. No correlation is evident within either the group of three crystalline fullerenes nor the group of 6 amorphous isomers.

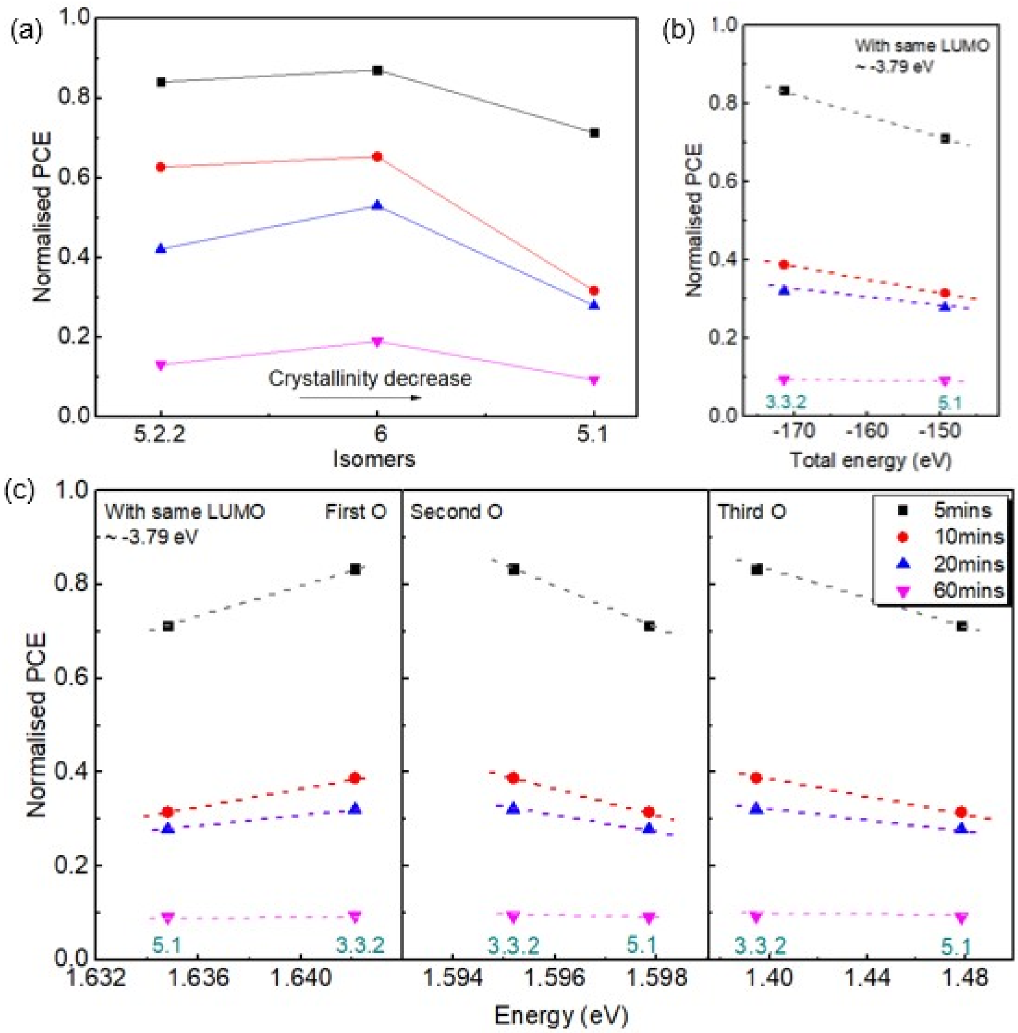

Fig. S7 Comparison of the PCE and molecular parameters in controlled group: (a) crystalline degree, (b) total energy with same LUMO level and crystallinity, (c) epoxidation energy with same LUMO level and crystallinity. Isomers 3.3.2 and 5.1 have the same LUMO level (~ -3.79 eV), while their degradation degree is different. Further comparison of isomers 3.3.2 and 5.1 we found that isomer 5.1 has a higher total energy of ~ -149 meV (~ -171 meV for isomer 3.3.2) and should be more active to facilitate the degradation. The analysis of the PCE and epoxidation energy for isomers 3.3.2 and 5.1 exhibits that isomer 3.3.2 only released higher energy than isomer 5.1 at the first epoxide position, while released less energy than 5.1 at other epoxide positions.

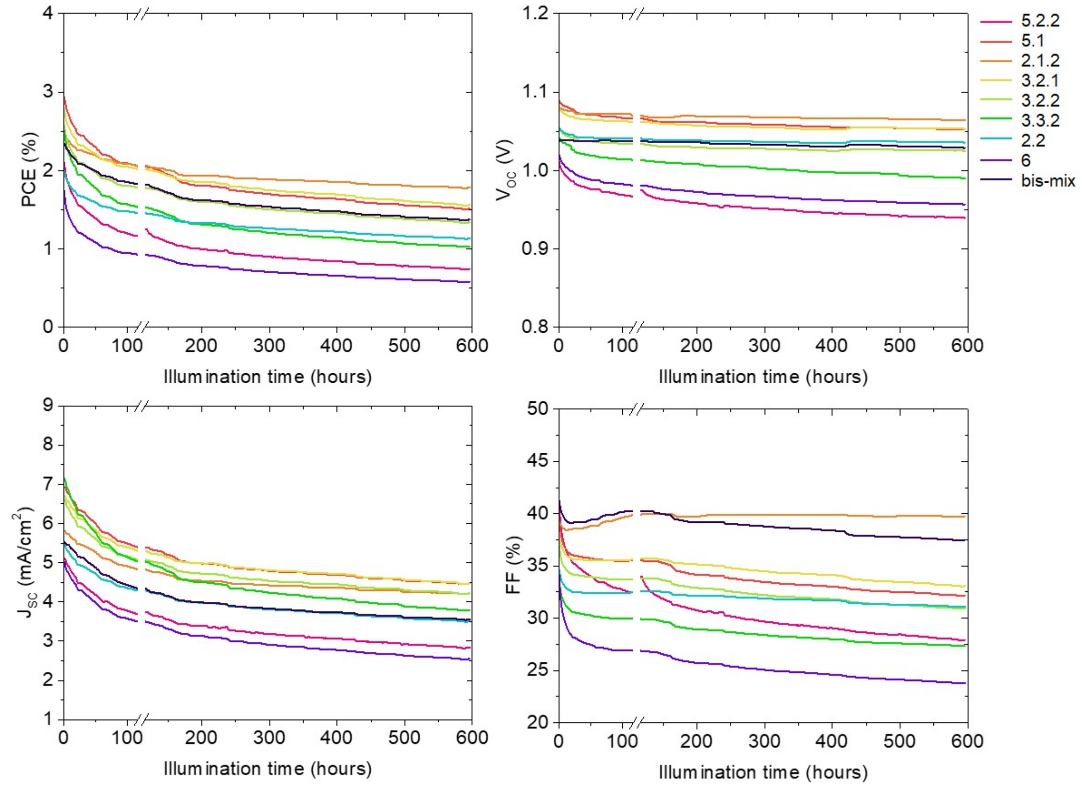

Fig. S8 The *J-V* characteristics of the burn-in degradation.

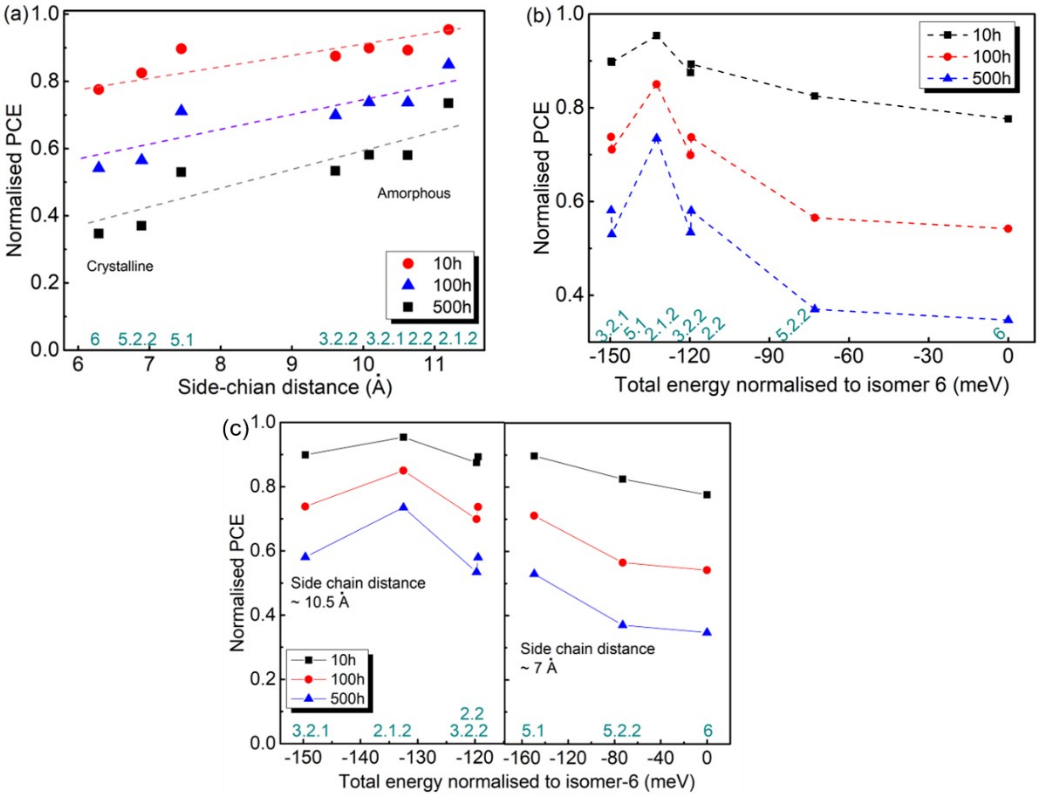

Fig. S9 Comparison between PCE and the fullerene molecular parameters of the burn-in degraded PCDTBT:bis-PCBM devices: (a) crystallinity and side-chain distance; (b-c) total energy.

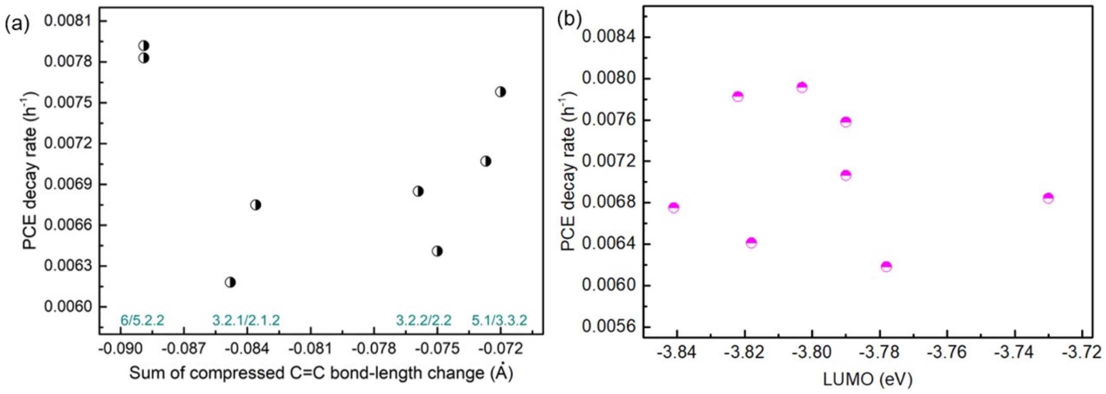

Fig. S10 Comparison between PCE decay rate and the fullerene molecular parameters of the burn-in degraded PCDTBT:bis-PCBM devices: (a) strain; (b) LUMO energy.

Table S1 The calculated the total energy of all potential PCBM molecules with one epoxide (PCBM-O). The bond lengths of 29 C=C bonds of PCBM (before epoxide formation) are measured in Gaussian software. The C atom labels are defaulted in Gaussian. The epoxide positions are classified into *cis*, *e* and *trans* groups. The calculations were done at the B3LYP level of theory with the 6-31g* basis set.

Bond	Epoxide total energy	Bond length	Group
11,12	-2977.1651991	1.38642	*cis*-1
7,8	-2977.1594464	1.38622	*cis*-1
13,14	-2977.1573108	1.3821	*cis*-1
5,6	-2977.1498295	1.38598	*cis*-1
24,25	-2977.1280249	1.39419	*e*
33,34	-2977.1257992	1.39417	*e*
55,56	-2977.1184535	1.39635	*trans*-2
16,17	-2977.1166114	1.39765	*cis*-2
20,38	-2977.1143046	1.39527	*e*
29,47	-2977.1142873	1.3953	*e*
27,28	-2977.1125238	1.3986	*cis*-3
48,49	-2977.1124708	1.39549	*trans*-4
15,32	-2977.1120019	1.39752	*cis*-2
21,22	-2977.1113878	1.39886	*cis*-3
30,31	-2977.1113460	1.39886	*cis*-3
10,26	-2977.1102709	1.39762	*cis*-2
39,40	-2977.1099343	1.39562	*trans*-4
9,23	-2977.1099056	1.39782	*cis*-2
45,46	-2977.1096586	1.39578	*trans*-4
36,37	-2977.1084433	1.39551	*trans*-4
57,58	-2977.1031124	1.39541	*trans*-1
61,62	-2977.1019122	1.39644	*trans*-2
59,60	-2977.1012425	1.39643	*trans*-2
53,54	-2977.0987649	1.39633	*trans*-2
41,42	-2977.0938023	1.39621	*trans*-3
43,44	-2977.0935814	1.39617	*trans*-3
50,51	-2977.0933131	1.39633	*trans*-3
35,52	-2977.0909695	1.39633	*trans*-3
18,19	-2977.0610033	1.39915	*cis*-3

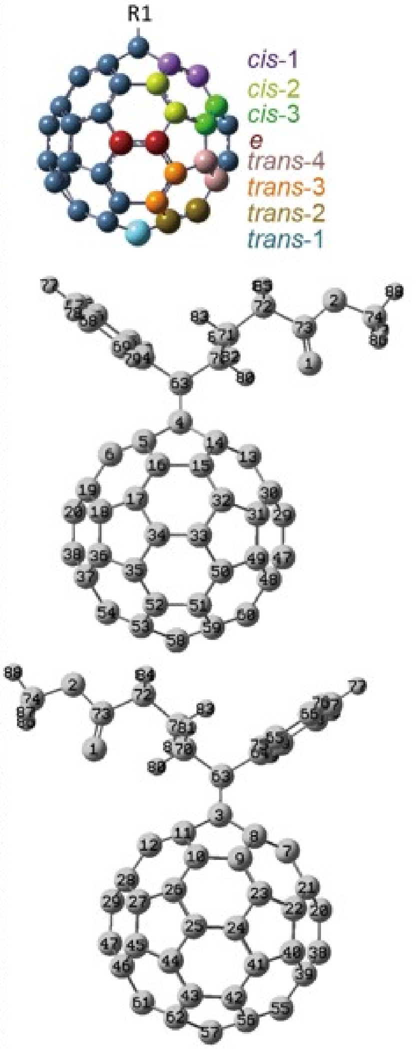

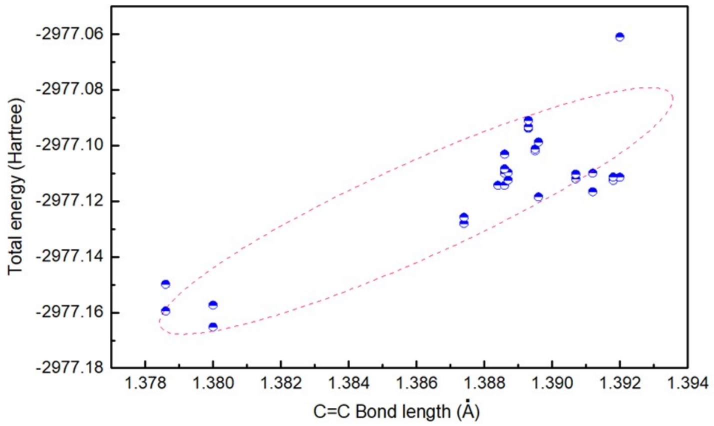

Fig. S11 Total energy *vs* C=C bond length plot, exhibiting a near positive relationship.

Table S2 Total energy of the PCBM-O and bis-PCBM-O epoxides obtained from Gaussian calculation after structural optimization with O at the *cis*-1 positions. The energy released during oxidation (epoxidation energy) was calculated by the method: $[E_T(\text{isomer})+ E_T(O_2)/2] - E_T(\text{epoxide})$. Total energy of oxygen $E_T(O_2) = -150.3113$ Hartree. The calculations were done at the B3LYP level of theory with the 6-311G (2df, 2pd) basis set.

	Isomer/Ha	O-1/Ha	O-2/Ha	O-3/Ha	O-4/Ha
PCBM	-2903.09052	-2978.69961	-2978.69765	-2978.69765	-2978.69087
2.1.2	-3519.91686	-3595.13236	-3595.13159	-3595.13057	-3595.12991
3.2.1	-3519.91749	-3595.13381	-3595.13268	-3595.13107	-3595.13099
2.2	-3519.91638	-3595.13211	-3595.13211	-3595.13040	-3595.13040
3.2.2	-3519.91639	-3595.13229	-3595.13044	-3595.13034	-3595.12964
3.3.2	-3519.91748	-3595.13429	-3595.13256	-3595.13120	-3595.13035
5.1	-3519.91829	-3595.13211	-3595.13075	-3595.12964	-3595.12834
5.2.2	-3519.91467	-3595.13191	-3595.13000	-3595.12840	-3595.12753
6	-3519.91199	-3595.13047	-3595.12809	-3595.12627	-3595.12442

	O-5/Ha	O-6/Ha	O-7/Ha	O-8/Ha
PCBM	-	-	-	-
2.1.2	-3595.12976	-3595.12905	-3595.12852	-3595.12778
3.2.1	-3595.13062	-3595.13004	-3595.12907	-3595.12877
2.2	-3595.12965	-3595.12965	-3595.12548	-3595.12548
3.2.2	-3595.12930	-3595.12845	-3595.12843	-3595.12683
3.3.2	-3595.13012	-3595.12636	-3595.12604	-3595.12519
5.1	-3595.12828	-3595.12783	-3595.12684	-3595.12636
5.2.2	-3595.12735	-3595.12616	-3595.12344	-
6	-3595.12263	-3595.11951	-3595.11869	-

Isomers	E_T /eV **released -1**	E_T /eV **released -2**	E_T /eV **released -8**
PCBM	12.3382	12.2848	12.1005
2.1.2	1.6117	1.5909	1.4871
3.2.1	1.6508	1.6202	1.5137
2.2	1.6346	1.5882	1.4543
3.2.2	1.6268	1.5764	1.4782
5.1	1.6348	1.5979	1.4785
3.3.2	1.6421	1.5952	1.3945
5.2.2	1.6759	1.6241	1.4454
6	1.7097	1.6450	1.3891

Table S3 The averaged device parameters from *J-V* characteristics of the photo-air degradation study. At least 6 pixels were measured for each device.

	Time/mins	PCE/%	V_{OC}	FF	J_{SC}/mAcm^{-2}
PCBM	0	4.59±0.2	0.93±0	0.59±0.007	8.3±0.33
	5	4±0.107	0.92±0	0.53±0.004	8.07±0.155
	10	2.95±0.131	0.885±0.001	0.445±0.005	7.6±0.192
	20	2.24±0.059	0.88±0.005	0.41±0.004	6.2±0.24
	60	1.1±0.071	0.78±0.01	0.397±0.007	3.72±0.282
mix	0	2.455±0.04	1.05±0	0.4±0.007	5.91±0.131
	5	1.265±0.021	1±0.003	0.35±0.005	3.61±0.068
	10	0.66±0.018	0.955±0.003	0.365±0.005	1.89±0.032
	20	0.59±0.03	0.945±0.004	0.35±0.003	1.77±0.068
	60	0.2±0.009	0.84±0.007	0.35±0.007	0.68±0.032
2.1.2	0	3.46±0.11	1.07±0.001	0.46±0	6.97±0.18
	5	2.72±0.054	1.05±0.005	0.433±0.008	6.07±0.1
	10	1.2±0.051	0.99±0.004	0.36±0.005	3.35±0.118
	20	1.47±0.021	1.01±0.005	0.335±0.004	4.37±0.081
	60	0.39±0.013	0.89±0.011	0.335±0.007	1.3±0.032
2.2	0	2.33±0.044	1.05±0.005	0.4±0.009	5.57±0.092
	5	1.315±0.028	1.02±0.004	0.34±0	3.85±0.112
	10	0.75±0.019	0.97±0.004	0.35±0.006	2.23±0.063
	20	0.955±0.039	0.99±0.005	0.33±0.005	2.92±0.11
	60	0.23±0.013	0.86±0.008	0.355±0.005	0.74±0.027
3.2.1	0	2.94±0.118	1.08±0.004	0.42±0.007	6.47±0.215
	5	2.2±0.1	1.05±0.005	0.365±0.006	5.78±0.128
	10	0.715±0.019	0.98±0	0.35±0.005	2.09±0.055
	20	0.82±0.038	0.985±0.007	0.345±0.004	2.41±0.047
	60	0.175±0.006	0.84±0.007	0.34±0.005	0.6±0.013
3.2.2	0	3.02±0.08	1.06±0.003	0.41±0.006	6.9±0.176
	5	2.01±0.118	1.025±0.003	0.355±0.007	5.53±0.212
	10	0.855±0.021	0.97±0.003	0.345±0.005	2.55±0.085
	20	0.79±0.032	0.97±0.004	0.32±0.005	2.515±0.098
	60	0.27±0.017	0.87±0.017	0.335±0.005	0.93±0.04
5.1	0	3.49±0.219	1.075±0.005	0.46±0.006	7.04±0.008
	5	2.91±0.205	1.06±0.005	0.405±0.017	6.73±0.302
	10	1.12±0.058	1±0.005	0.355±0.007	3.15±0.13
	20	1.355±0.087	1.02±0.005	0.345±0.005	3.87±0.281
	60	0.33±0.023	0.875±0.027	0.33±0.008	1.16±0.089
3.3.2	0	2.835±0.064	1.06±0.005	0.41±0.008	6.51±0.123
	5	2.02±0.066	1.03±0.006	0.34±0.004	5.75±0.24
	10	0.79±0.017	0.97±0.005	0.355±0.005	2.3±0.057
	20	0.895±0.022	0.99±0.004	0.35±0	2.56±0.038
	60	0.26±0.071	0.83±0.005	0.33±0.004	0.95±0.155
5.2.2	0	2.79±0.156	1.03±0.005	0.46±0.009	6.19±0.32

	5	2.34±0.053	1.01	0.43±0.01	5.65±0.095
	10	1.171±0.121	0.955	0.345±0.004	3.66±0.18
	20	1.746±0.176	0.98	0.375±0.009	4.97±0.36
	60	0.362±0.013	0.84	0.34±0.004	1.34±0.035
6	0	2.71±0.129	1.02	0.46±0.008	5.85±0.215
	5	2.356±0.059	1.01	0.44±0.006	4.9±0.12
	10	1.767±0.037	0.973	0.4±0.005	3.86±0.098
	20	1.43±0.055	0.973	0.342±0.004	3.27±0.127
	60	0.51±0.11	0.8326	0.33±0.008	1.58±0.069
